# Detection of Microorganisms Using Graphene-Based Nanobiosensors

**DOI:** 10.17113/ftb.59.04.21.7223

**Published:** 2021-12

**Authors:** Mehrab Pourmadadi, Fatemeh Yazdian, Sara Hojjati, Kianoush Khosravi-Darani

**Affiliations:** 1 School of Chemical Engineering, College of Engineering, University of Tehran, Tehran, Iran; 2 Department of Life Science Engineering, Faculty of New Science and Technologies, University of Tehran, Tehran, Iran; 3 Department of Food Technology Research, National Nutrition and Food Technology Research Institute, Faculty of Nutrition Sciences and Food Technology, Shahid Beheshti University of Medical Science, P. O. Box 19395-4741, Tehran, Iran

**Keywords:** graphene, graphene oxide, reduced graphene oxide, graphene quantum dots, microorganism detection, nanobiosensors

## Abstract

Having an insight into graphene and graphene derivatives such as graphene oxide, reduced graphene oxide and graphene quantum dots is necessary since it can help scientists to detect possible properties and features that could be useful when using these carbon materials in preparation of a nanocomposites. In recent years, graphene and its derivatives have attracted a lot of attention and been extensively applied in biosensors due to fascinating properties, such as large surface area, optical and magnetic properties, and high elasticity for the detection of microorganisms as they can be modified with some other materials such as macromolecules, oxide metals and metals to improve the electrochemical behaviour of the biosensor. In this review paper, biosensor design strategies based on graphene and its derivatives (graphene-based nanocomposites in biosensors) are described. Then their application for the detection of microorganisms including prions, viroids, viral and bacterial cells as well as fungi, protozoa, microbial toxins and even microbial sources of antibiotics is reviewed.

## INTRODUCTION

Graphene is a monolayer of carbon atoms, arranged in a honeycomb lattice. Each of these carbons participates in three intralayer sp^2^ or sigma (σ) bonds with its three neighbouring carbon atoms (*1*). Although these bonds, known as covalent bonds, make this graphene layer very strong, this strength is still limited by the presence of defects and grain boundaries (*2*). In addition to a monolayer graphene, bi-, few- and multilayer graphene exists as well. One- to <10-layer graphene is called a 2D crystal, while a structure consisting of a higher number of graphene layers is considered a 3D thin film (*3*). Interlayer pi (π) bonds between two graphene layers or between graphene and other molecules are usually weaker than sigma bonds and are responsible for electrical and thermal conductivities and functional group attachments which is important in sensor applications (*4*). Graphene oxide (GO) as a nanomaterial obtained by the chemical peeling of graphite using strong oxidizing agents can be modified with some other materials such as macromolecules, metal oxides and metals to improve the electrochemical behaviour of the biosensor (*5*, *6*). Graphene-based nanocomposites in sensors have received significant attention (*6*). Functional groups, such as hydroxyl and epoxy, are present in the base plate, as well as carboxyl, carbonyl and phenol at the GO edge. Compared to graphene, GO shows different optical, electrical and electrochemical behaviour due to its oxygen-containing structure (*7*), and has been considered as a promising material in biotechnology (*5*). Fourier-transform infrared (FTIR) analysis offers detailed information on GO structure, *e.g.* absorption bands at 3360 and 1040 cm^−1^ corresponding to OH and C-O groups, respectively. Furthermore, an absorption peak at 1710 cm^−1^ is related to C=O functional groups that can react with the functional groups of other biomaterials, such as aptamer chains. The synthesis and characterization of targeted delivery using chitosan-magnetite-reduced graphene oxide is possible as nanocarrier (*8*).

In this review paper, we focus on the biosensor design strategies based on graphene and its derivatives (graphene-based nanocomposites) due to their attractive properties for microorganism detection.

## GRAPHENE-BASED NANOCOMPOSITES IN BIOSENSORS

Fascinating properties of graphene, such as large surface area, optical and magnetic properties, and high elasticity, make it an appropriate basic structure for preparing graphene-based nanocomposites (*9*). Depending on the number of graphene layers, the absence or presence of defects, the materials used in combination with graphene, and what kind of assembly methods are used, several nanocomposites with different features, electrochemical properties and applications have been reported (*10*).

Normally graphene tends to agglomerate through van der Waals and π-π stocking bonds, so various methods have been proposed to solve this problem (*11*). It has been shown that hybridizing metal nanoparticles with graphene sheets is electrically conductive and improves the heat of graphene. Hybridization also prevents aggregation by creating gaps between graphene sheets (*12*). Gold nanoparticles with unique properties have a great potential to form hybrids with graphene and create a new structure with many applications in electrochemistry (*13*). In electrochemical sensors, gold nanoparticles can increase the sensitivity of the sensor for pathogen detection (*14*). Nanoparticles may have the role of catalyst in electron transfer between the analyte and the electrode surface, so fabrication of nanomaterial-based biosensor has been reported for measurement of a microRNA involved in cancer (*15*). On the other hand, graphene itself plays an important role in increasing the speed of electron transfer. The presence of oxygen groups on graphene layers has a great effect on the adsorption and surface desorption of chemical reaction products from the surface of graphene electrodes. In a report, carbon paste electrode coated with nano-graphene-platelet/Brilliant-green composite was used for electrocatalytic oxidation of flavanone hesperidin (*16*). Adsorbed products often slow down the electrochemical reaction for highly sensitive compounds to oxygenated groups, in this regards gold nanoparticles-reduced graphene oxide-based electrochemical immunosensor can be used for the detection of cardiac biomarker myoglobin (*17*). The graphene oxide layer, which has oxide edges, is placed vertically or obliquely between the electrode surface and the active centre of the biomarker (*18*). Studies have shown that gold graphene nanohybrid-based biosensor has increased biocompatibility and measurement sensitivity, which can be applied for cholesterol biosensing (*19*). Some of these nanocomposites are described in this review. Using other carbon nanomaterials with graphene is a good way to increase its novel properties due to their synergistic effects and make new more efficient composites than each of the carbon nanomaterials individually. These properties include electrochemical activity, electrical conductivity, large surface area, ease of functionalization and biocompatibility (*20*). Variation in the structure and compatibility of chemical properties make different carbon nanomaterials such as graphene, carbon nanotubes, fullerene, nanodiamonds, *etc*. appropriate hybrids to form different possibilities of binding to various recognition agents in a biosensor system (*7*). An example of this effective synergistic action is shown by Liu *et al.* (*21*), where the addition of GO or carbon nanotubes (3%, by mass) improved twofold the tensile strength of polyelectrolyte complex (PEC) membranes.

Metal nanoparticles, such as Au-, Pt-, Pd-, Ag- and Li-nanoparticles as well as their oxide and sulfide compounds, are frequently used in combination with graphene to form favourable nanocomposites in different types of biosensors. Due to their free electrons, metal nanoparticles can absorb visible and ultraviolet light, and therefore are applicable in many optical biosensors using surface plasmon resonance effect (*22*). The adequate catalytic properties of metal nanoparticles make them ideal for electrochemical biosensors, *e.g*. as probe oligonucleotide immobilization platform in a DNA biosensor (*23*). On the other hand, large surface area, great mechanical strength and electrostatic adsorption of biomolecules are the main properties of metal nanoparticles that are useful for sensing bacteria (*22*). Govindhan *et al.* (*24*) reported that more pronounced anodic peak in the cyclic voltammograms is obtained with Au/reduced graphene oxide (RGO)/glassy carbon electrode (GCE) than with Au/GCE or RGO/GCE electrodes, confirming that RGO and GCE have better electrochemical properties when used together.

When designing an efficient scaffold, there have to be agents to recognize the target microorganism or its product. Choosing an appropriate agent is of high importance since it has direct influence on the results of all evaluation criteria of a biosensor, such as the limit of detection (LOD), linear range of detection, detection time, selectivity, reliability and reproducibility. Biorecognition elements provide specificity, selective and strong affinity to the targets (*25*). They may be natural, such as enzymes, antibodies and nucleic acids; pseudo-natural, such as aptamer; or synthetic, such as molecularly imprinted polymers (MIPs). Nucleic acids, peptides, proteins, antibodies and phages are more or less used as biorecognition elements to detect microorganisms or their products. Criteria for selection of the type of recognition element and methods of their immobilization on graphene could offer ideas to be used in the manufacture of other biosensors with different target elements as well (*26*). DNA-graphene hybrids that are mainly prepared by self-assembly induced by ultrasonication are supposed to match a certain sequence of the genome (*27*).

Zhang *et al.* (*23*) prepared a graphene-pyrenebutyric acid nanocomposite by ultrasonication and covalently immobilized amino-modified oligonucleotides on the nanocomposite through linkage with carboxylic groups of pyrenebutyric acid.

A bacteriophage is a virus that recognizes its specific receptors on bacteria and archaea. Phages can infect these cells and, through different steps, replicate themselves within them. With the aid of immobilized peptides or proteins on their surface, phages can bind to a vast range of molecules. Bhardwaj *et al.* (*28*) succeeded in covalent immobilization of bacteriophages specific for bacteria *Staphylococcus arlettae* on a carboxylated graphene surface due to bonds between carboxyl groups of graphene and –NH_2_ groups of the bacteriophage head. In other words, bacteriophages are useful in the phage display to carry a certain gene and represent the peptide that belongs to this gene on their surface. This method helps to find the correlation between specific genotypes and their unknown phenotypes, besides finding peptides that can bind to a particular target (like amyloid beta oligomer) and could be used as recognition elements in biosensors (*29*). Aptamers and different types of receptors, such as enzymes and antibodies, and other biorecognition elements can be immobilized through covalent and non-covalent bonds (*30*). Immobilization is done either chemically or physically by interacting or trapping receptors, respectively. It is one of the most demanding steps in designing a sensor. The choice of the appropriate method for immobilization depends on the nature and physicochemical conditions of the transducers and receptors (*26*). Entrapment, microencapsulation, sol-gel technique and adsorption belong to physical immobilization methods and are mostly used for sensors that have enzyme receptors. Another method is chemical immobilization, which is usually based on creating a chemical bond between the functional groups on the surface of the transducers and the receptors (*31*). It usually occurs through cross-linking chemical reagents such as glyoxal, hexamethylenediamine, glutaraldehyde, carbodiimide, *etc*. Cross-linking is part of the covalent binding that is usually accomplished by activating amine and carboxyl functional groups, which results in strong, highly stable and effective binding. Pure graphene, as mentioned, can prepare a charged region for the adsorption of any charged molecules or metal ions as an interaction in empty dots. Graphene derivatives are synthesized by their oxide components due to the synthesis of large amounts of epoxy, hydroxyl and carboxyl groups at the edges and surfaces. The active (functionalized) region of graphene is able to directly bind to heteroatoms, nanoparticles (NPs), enzymes, antigens, proteins, antibodies, DNA and other specific molecules (*25*). Graphene can also increase sensitivity and LOD of a biosensor device by improving the charge or electron transfer between the graphene and the biomolecules due to its extraordinary properties (*32*).

## DETECTION OF MICROORGANISMS

The direct and indirect effects of microorganisms and their products on human health are of great concern for both governments and societies globally. Many of the microorganisms spread in the air, water, soil, food, plants and animals are beneficial or even vital for human existence, so it is necessary to distinguish harmful microorganisms from the safe ones and determine their concentration in different types of samples. Today, many fields of research, such as environment, food safety and health care, are working on developing new methods and more efficient devices for such a purpose. Among them, the design and the production of more cost-effective biosensors with better selectivity, sensibility and stability are of particular importance. Given the excellent properties of the graphene, it is evident that this nanostructure is a great candidate for use in the field of biosensing. Next chapters review graphene-based biosensors for the detection of each group of microorganisms and microbial products.

### Detection of prions

Prions are misfolded proteins that can cause several neurological diseases in humans and animals (*33*). The main reason for the misfolding of the structure of proteins and their conversion to prions is not clear. This abnormal three-dimensional structure causes infections, protein-misfolding diseases and protein collapses. Prions formed by the aggregation of abnormal proteins are called amyloids, which are the main cause of diseases such as Alzheimer's and Parkinson's (*33*, *34*). Liu *et al.* (*35*) constructed a GO-based fluorescent biosensor for the selective measuring of amyloid-β oligomer concentration. Zhao *et al.* (*30*) developed a complex of GO-Au nanoparticle-aptamer for amyloid-β oligomer detection using ELISA immunoassay. They applied a sandwich aptamer-Aβ oligomer-antibody to assist in the detection of prions at 50 pM. Lou *et al.* (*36*) reported surface plasmon response detection of prion disease-associated isoform (PrP^Sc^) applying aptamer-graphene oxide and the results showed a good linearity in the concentration range of about 0.001–1 ng/mL. Zhuang *et al.* (*37*) designed resonance energy transfer sensitive biosensor for prion protein by using graphene oxide and aptamer beacon and the results show good linearity between 10.2 and 78.8 μg/mL with a detection limit as low as 0.309 μg/mL and high selectivity. Zhou *et al.* (*38*) obtained an Au-vertical graphene/carbon cloth electrode for applying poly(thymine)-templated copper nanoparticles as probes for ultrasensitive detection of amyloid-β oligomer. This biosensor showed a low detection limit of about 3.5 pM and excellent specificity with great stability.

### Detection of viroids

Viroids are classified as single-stranded RNA with no protein covering. Many viruses, such as HIV, Epstein-Barr, human cytomegalovirus, Ebola, human herpesvirus, hepatitis C or dengue, can encode unique viral MiRNAs that are critical to transcription mechanisms of gene expression and viral replication (*39*). MiRNAs are non-coding sequences of 20-25 nucleotides. Therefore, the identification of viroids and miRNAs is of great importance in clinical diagnoses. Low *et al.* (*40*) created a graphene/ZnO/PSE-modified electrochemical impedance genosensor with enhanced sensitivity properties for detection of coconut cadang-cadang viroid. Malecka *et al.* (*41*) developed an electrochemical genosensor using screen-printed gold electrodes for specific DNA and RNA sequences derived from avian influenza virus H5N1. This method was able to detect approx. 280-mer RNA sequences.

### Detection of viral cells

Since viruses cause many diseases in humans, animals and plants, especially viruses that are detrimental for human health such as HIV (*42*), hepatitis A, B (*43*) and C (*44*), human cytomegalovirus (*45*), Ebola or human herpesvirus (*46*), the detection of viruses is clinically crucial (*47*). Navakul *et al.* (*48*) proposed a novel approach to the diagnosis of dengue virus and antibody screening using an electrochemical biosensor based on graphene polymer. A reduced graphene oxide-based field-effect transistor for immunodetection of Ebola virus was reported with a limit of detection as low as 2.4 pg/mL (*45*). Singh *et al.* (*49*) developed an electrochemical immunosensor integrated with a microfluidic platform applying a reduced graphene oxide for influenza virus detection that exhibited good selectivity and an enhanced detection limit expressed in plaque forming units (PFU) of 0.5 PFU/mL, and a high linearity of H1N1 virus in the concentration range of 1 to 104 PFU/mL (R^2^=0.99).

### Detection of bacterial cells

Graphene-based nanosensors have been reported for rapid and sensitive detection of bacteria (*50*-*56*). A bacterium can be observed as a whole cell whether it is active or inactive. In many cases, it is important to distinguish between these two. For instance, to evaluate a particular antibacterial treatment, it is necessary to compare the concentration of the bacterial population within the sample before and after the treatment. In the case of detecting a whole cell, it is more common to use an antibody or aptamer, which is specific for a certain antigen on the bacterium surface (*52*), or use a phage of which the bacterium of interest is the host. Muniandy *et al.* (*51*) developed an electrochemical aptasensor based on a reduced graphene oxide-titanium dioxide nanocomposite for detection of *Salmonella enterica* and the optimized aptasensor showed high sensitivity with a wide detection range (10-10^8^ CFU/mL), and also a low LOD of 10 CFU/mL for *Salmonella* sp. Singh *et al.* (*53*) developed a microfluidic immunochip applying biofunctionalized graphene oxide for *Salmonella* sp. detection with the LOD as low as 0.376 CFU/mL. Chang *et al.* (*50*) reported ultrasound-assisted self-assembly of monolayer graphene oxide with a high affinity for *Escherichia coli* with LOD as low as 10 CFU/mL, a highly sensitive and selective field-effect transistor. Dehghani *et al.* (*52*) made a graphene oxide and graphene dot-based fluorescence resonance energy transfer biosensor for immunosensing of *Campylobacter jejuni* and the results showed a good LOD for these bacteria of about 10 CFU/mL. Pandey *et al.* (*54*) developed a graphene-based electrical biosensor for the detection of pathogenic *E. coli* O157:H7 in food which showed sensitivity as low as 10–100 cell/mL. Hernández *et al.* (*55*) reported a potentiometric biosensor for living bacterium detection based on graphene, which could detect a single CFU/mL of *Staphylococcus aureus* with a very low time of detection.

### Detection of fungi

Because of their elaborate genetic makeup and metabolism, fungi are considered geological microorganisms (*57*). In addition, a group of microorganisms plays an important role in the environment, agriculture, forestry and human health. In ecology, fungi play a role as a biosphere balance. They are the main source of antibiotic production, and among the many species of fungi, *Aspergillus* spp. has attracted the most attention. For example, there are several fungal plant pathogens, which can cost billions of dollars a year in crop damage. Fungi also affect humans by contaminating and spoiling food (*58*-*62*). Qi *et al.* (*60*) developed an electrochemical biosensor applying impedance methods based on graphene-Au nanoparticles for *Aphanomyces invadans* detection. As discussed, graphene and graphene derivatives such as GO, reduced GO and graphene quantum dot nanocomposites are promising nanomaterials that can be used for fungal detection.

### Detection of protozoa

Protozoa are a group of single-celled eukaryotes that may be free living or parasitic. Some protozoa have a two-phase life cycle, alternating between proliferative stages (such as trophozoites) and dormant cysts. Historically, protozoa have been categorized as single-celled species, distinct from phototaxis, single-celled photosynthetic organisms (algae) that are called primitive plants. In both classes, the rank of phylum was commonly granted under the Protista kingdom. Jain *et al*. (*63*) suggest that oocyst of *Cryptosporidium parvum* can be used as a template for the assembly of nanomaterials due to its interaction with gold nanoparticles and GO.

## DETECTION OF MICROBIAL TOXINS

Besides the microorganisms themselves, their secondary metabolites could lead to unwanted consequences for human health, mainly because of food spoilage or water contamination, and as a result, cause different diseases. These concerns are the main reasons for seeking new and effective methods for detecting these hazards. One of the main groups of microbial toxins is those produced by fungi. Mycotoxins are a range of fungal toxins that can contaminate raw and processed foods during different steps of preparation. They can be determined by graphene-based nanosensors (*64*-*66*). As a very stable compound and the most occurring mycotoxin, ochratoxin A is produced by *Aspergillus ochraceus, Aspergillus carbonarius and Penicillium verrucosum*. This toxin may be present in many daily consumed foods. Ochratoxin A may induce apoptosis in several cell types or may increase the incidence of tumours in humans. PVP-coated gold nanoparticles have been reported for the selective determination of ochratoxin A *via* quenching fluorescence of the free aptamer (*64*).

Aflatoxin is a widely present mycotoxin produced by *Aspergillus flavus* in both plant and animal food products (*67*, *68*). This group of mycotoxins consists of four main subgroups, namely aflatoxin B_1_, B_2_, G_1_ and G_2_. Aflatoxin B_1_ is believed to have the biggest role in causing liver cancer among all other groups of aflatoxins. Aflatoxin M_1_ is another subgroup that is mostly known to be present in dairy products and even breast milk of lactating mothers. Although the toxicity of aflatoxin M_1_ is ten times lower than of the B_1_ subgroup, consuming this toxin at a very early age may cause impaired growth, especially in infants, who are much more vulnerable to any harm (*68*).

Zearalenone is a mycotoxin produced by *Fusarium* species, which is frequently present in cereal grains and animal feeds (*69*). This mycotoxin is mainly known for its xenoestrogenicity, which means having a similar structure to estrogen and, therefore, a great affinity to attach to the estrogen receptors. This activity has been shown to cause reproductive disorders like the low quality of semen and hormone imbalance in mice and is carcinogenic for humans, causing endometrial or breast cancer.

Besides fungi, numerous other microbial cells are capable of producing harmful toxins. Microcystin is produced by cyanobacteria and it contaminates water. This toxin can induce cancer, especially liver tumour, due to its inhibitory effect on certain protein phosphatase activities (*70*, *71*).

An example of toxins produced by bacteria is a polypeptide called the cholera toxin of the bacterium *Vibrio cholerae*. First, this toxin binds to the ganglioside GM1 of the target cell membrane and continues a process that leads to activation of adenylate cyclase and promotes secretion of water and ions into the intestinal lumen, ending with severe diarrhoea (*72*). Drinking sewage-contaminated water or consuming crops cultivated with this water are some ways of vibrio transmission into the human body. An electrochemical biosensor has been developed for the rapid detection of cholera toxin based on air-stable lipid films with incorporated ganglioside GM1 using graphene electrodes.

Enterotoxins are another group of bacterial toxins produced by *Staphylococcus aureus.* Biosensor detection of botulinum toxoid A and staphylococcal enterotoxin B in food has been reported (*73*). These toxins are made of protein and are mostly heat-stable. Enterotoxin type B, as an example, is produced by *Staphylococcus aureus* and can cause diarrhoea as a result of consuming contaminated foods, which range from milk and cheese to ham and sausages. Contamination can be due to the bacterium favourable growth temperatures in processing steps. Botulinum is another bacterial toxin that is produced by the bacterium *Clostridium botulinum* and causes food poisoning in addition to its possibility of being used as a bioterrorism tool. To prevent deadly results of consuming this neurotoxin, very accurate methods are needed to detect the toxin on the scale of nanograms, especially in canned food products.

## DETECTION OF MICROBIAL SOURCES OF ANTIBIOTICS

The consumption of food products such as meat, milk, honey and vegetables or pharmaceutical products containing antibiotics causes accumulation of this metabolite in the human body, which could lead to different types of diseases (*74*-*80*). Chloramphenicol is an example of an antibiotic used against Gram-positive and Gram-negative bacteria, also used as a veterinary drug and even water disinfectant due to its low cost and effectiveness. However, it may cause potential side effects such as the development of plastic anemia, a blood disorder, and the failure of bone marrow to produce blood cells mainly because of its toxic transformation by-products (*74*). By disrupting mitochondrial iron metabolism, chloramphenicol causes problems with the iron-sulfur clusters (FeS) of the electron transport chain, especially depletion of adenosine triphosphate (ATP), which probably leads to tumourigenesis (*75*). It has been shown that chloramphenicol residues have long persistence of at least 35 days after the end of the treatment in animal tissues (*76*). Metronidazole has antibacterial and anti-inflammatory effects, which makes this antibiotic part of protozoal disease treatments, but its excessive long-term usage may be genotoxic, carcinogenic and mutagenic (*77*, *78*). Neomycin is an aminoglycoside antibiotic found in eardrops with possible ototoxic properties, which can cause hearing impairment or loss by inducing auditory hair cell apoptosis. Although bleomycin has an essential advantage as an antitumour antibiotic in many anticancer drugs, overuse of it may have a toxic effect on the lungs, which leads to pulmonary dysfunction and, subsequently, death (*79*). Oxytetracycline, which is widely used in dermatology and veterinary medicine, can decrease melanocyte viability, relative to the drug dosage (*78*). Streptomycin has been broadly used in veterinary drugs and pesticides to control different groups of microorganisms. A high amount of this antibiotic in food products has the potential to cause ototoxicity and nephrotoxicity (*80*). For all those reasons, it is of outmost importance to develop a fast method for determination of antibiotic residues in food (*77*, *78*, *80*). In Table 1 (*5*, *23*, *35*, *36*, *40*, *49*, *60*, *77*, *81*-*95*), a comprehensive list of graphene-derived materials that have been applied as biosensors for detection of microbially derived antibiotics, as well as prions, viroids, viruses, bacterial cells, fungi, protozoa and microbial toxins, is given.

**Table 1:** Graphene and its derivative materials used as biosensors for detection of prions, viroids, viruses, bacterial cells, fungi, protozoa, microbial toxins and microbially derived antibiotics

Graphene and its derivatives	Materials in composition with graphene	Biorecognition element	Detected material	Detection limit	Linear range	*t*(detection)/min	Type of biosensor	Ref.
*Detection of prions*								
GO	-	FITC-PrP(95–110)	amyloid-β oligomers	-	0.01–2 µM	60	fluorescent	(*35*)
GO	-	ssDNA	sensitive prion disease-associated isoform	4.2410^−5^ nM	0.001–1 ng/mL	40	surface plasmon resonance	(*36*)
*Detection of viroids*								
Graphene	zinc oxide	ssDNA	coconut cadang-cadang viroid	4.310^–12^ M	10^–11^–10^−6^ M	60	electrochemical	(*40*)
GQD	SiO_2_ nanoparticles and NaYF_4_:Yb,Er	ssDNA	miRNA HIV1-miR-Tar-5p	10 fM	above 10^-6^ M	-	fluorescent	(*81*)
*Detection of viruses*								
GO	1-pyrenebutyric acid *N*-hydroxysuccinimide ester	antibody	rotavirus	10 PFU	10–10^5^ PFU/mL	-	electrochemical	(*23*)
								
Graphene	poly(3-thiophene boronic acid) and gold nanoparticles	antibody	avian leukosis viruses	210 tissue culture infective dose per 50 mL	527–3162 infective dose per 50 mL	-	electrochemical	(*82*)
Graphene	silver nanoparticles and chitosan	H7-polyclonal antibody	avian influenza virus H7	1.6 pg/mL	1.610^-3^–16 ng/mL	30	electrochemical	(*83*)
RGO	-	antibody	rotavirus	10^2^ PFU	10–10^5^ PFU/mL	-	field-effect transistor	(*84*)
GO	-	-	enteric EV71 and H9N2	-	-	30	RT-PCR	(*85*)
GO	-	ssDNA	Ebola virus	1.4 pM	30 fM–3 nM	-	fluorescent	(*86*)
RGO	-	antibody	influenza virus H1N1	10^2^ PFU	10–10^6^ PFU/mL	15	electrochemical	(*49*)
RGO	MOS_2_	ssDNA	human papillomavirus	0.1 ng/mL	0.2–2 ng/mL	-	electrochemical	(*87*)
*Detection of bacterial cells*								
GNPs	SiO_2_ substrates- graphene (GNPs and Mg)	anti-*E. coli* antibodies	*E. coli* O157:H7	10–100 cell/mL	10^2^–10^6^ (GNPs) and 10–10^7^ cell/mL (GNPs+Mg)	30	electrical	(*88*)
Graphene	carboxyl	a virulent phage called PaP1	*Pseudomonas aeruginosa*	56 CFU/mL	1.4·10^2^−10^6^ CFU/mL	30	electrochemiluminescent	(*89*)
GO	-	anti-*E. coli* β-gal Abs	*E. coli*	10–100 μg/mL	-	-	infrared spectroscopy	(*90*)
QDs and GO	-	complementary to the *invA* oligo	*Salmonella*-specific *invA* gene	4 nM	-	20	fluorescence resonance energy transfer	(*91*)
GQD	nitrogen-doped GQD	*E.* *coli* polyclonal antibody	*E. coli* O157:H7	8 CFU/mL	10–10^7^ CFU/mL	120	ECL	(*92*)
RGO	indole-5-carboxylic acid	ssDNA	*Klebsiella pneumonia*	target DNA down to 310^-11^ M	10^-6^–10^-10^ M		electrochemical	(*93*)
RGO	RGO-Cu(II)	monoclonal antibodies	*Staphylococcus aureus*	4.4 CFU/mL	10–10^8^ CFU/mL		electrochemical	(*94*)
*Detection of fungi*								
Graphene	gold nanoparticles and cysteamine	antibody (anti-mycelium)	*Aphanomyces* *invadans*	309 ng/mL	0.2–4 mg/mL	90	electrochemical	(*60*)
*Detection of microbial toxins*								
RGO	gold nanoparticles	ssDNA	endotoxin	1 fg/mL	0.1–0.9 pg/mL	30	electrochemical	(*5*)
Graphene	-	antibody	microcystin-LR	0.05 μg/L	0.05–20 μg/L	-	electrochemical	(*95*)
*Detection of microbially derived antibiotics*								
Graphene	silver nanoparticles/sulfonate	-	chloramphenicol	0.01 mM	0.02–20.0 μM	-	electrochemical	(*77*)

GO=graphene oxide, GQD=graphene quantum dots, RGO=reduced graphene oxide, GNPs=graphene nanoparticles, RT-PCR=reverse transcription polymerase chain reaction, ECL=electrochemiluminescence

## CONCLUSIONS

In this review, we presented a comprehensive point of view on the intrinsic properties and application of graphene and graphene derivatives in microorganism detection using graphene-based nanobiosensors. Recently, graphene has become a well-known 2D nanomaterial and graphene derivatives such as graphene oxide (GO), reduced GO and graphene quantum dot nanocomposites have fascinating properties, including large surface area, optical and magnetic properties, and high elasticity, which makes it an appropriate basic structure for preparing several graphene-based nanocomposites. They are scaffolds for immobilizing biomolecules and create highly selective biosensors. Based on recent studies, among several detection methods applying graphene-based nanobiosensors, the most common is electrochemical one due to its simplicity and high sensitivity in a rapid assay. Due to these attractive properties and features, these carbon structures can be used in biosensors for the detection of microorganisms such as prions, viroids, viral cells, bacterial cells, protozoa, microbial toxins, fungi and antibiotics from microbial sources, among others.
